# Drosophila melanogaster models for investigating inflammatory bowel disease: Methods, pathology, mechanisms, and therapeutic approaches

**DOI:** 10.17305/bb.2025.12656

**Published:** 2025-07-01

**Authors:** Xinyi Li, Shushen Sun, Xiaoxi Liu, Qinghao Meng, Mengzhe Tian, Jingyi Li, Suxia Ren, Zengyi Huang, Yiwen Wang, Shaoshan Du

**Affiliations:** 1 School of Pharmaceutical Science and Technology, Faculty of Medicine, Tianjin University, Tianjin, China; 2 Department of Gastroenterology, Tianjin University Jinnan Hospital, Tianjin, China; 3 First Teaching Hospital of Tianjin University of Traditional Chinese Medicine, Tianjin, China; 4 School of Basic Medical Sciences, Chongqing Medical University, Chongqing, China; 5 National Clinical Research Center for Child Health and Disorders, Children’s Hospital of Chongqing Medical University, Chongqing, China

**Keywords:** Inflammatory bowel disease, IBD, *Drosophila melanogaster*, gut microbiota, intestinal stem cells, ISCs, inflammatory pathways, natural products

## Abstract

Inflammatory bowel disease (IBD) is a complex disorder characterized by chronic gastrointestinal inflammation. This paper examines the use of Drosophila melanogaster as a model organism to investigate interactions among the gut microbiota, intestinal stem cells (ISCs), and signaling pathways involved in IBD pathogenesis. Key findings indicate that dysbiosis of the gut microbiota significantly contributes to IBD by altering immune responses and inflammatory signaling, leading to increased intestinal damage. Additionally, ISCs are crucial for intestinal regeneration; their dysregulation exacerbates injury, highlighting their role in maintaining gut homeostasis. Natural compounds, particularly those derived from traditional herbal medicines, show promise in alleviating IBD symptoms by targeting oxidative stress, regulating inflammation, and modulating autophagy, thus promoting ISC homeostasis and restoring microbial balance. This review underscores the intricate relationships among the gut microbiota, ISCs, and inflammatory pathways in IBD, as elucidated through Drosophila studies. The studies summarized here emphasize the need to address microbial imbalances, ISC dysregulation, and inflammatory mechanisms to develop effective therapeutic strategies. Further research is essential to fully elucidate these interactions and inform innovative treatments that improve patient outcomes in IBD management.

## Introduction

Inflammatory bowel disease (IBD) is a chronic, non-specific inflammation affecting the gastrointestinal tract, primarily comprising ulcerative colitis and Crohn’s disease [1]. Historically, IBD has been more prevalent in economically developed regions, such as North America and Northern Europe. However, with global westernization, especially the spread of Western diets, the incidence of IBD in emerging industrialized countries in Asia and Latin America has significantly increased since the early 21st century [2]. Globally, the number of IBD patients rose significantly from 3.3 million in 1990–4.9 million in 2019 [3], with projections indicating that it could exceed 10 million by 2030 [4].

IBD has a protracted course, high disability rates, and necessitates long-term medication. Therefore, IBD patients face substantial medical costs [5]. The rising incidence, coupled with substantial treatment costs, has created a significant economic burden on society. Furthermore, the gut interacts intricately with other organs, such as the brain and lungs, through the brain-intestinal axis and lung-intestinal axis. Consequently, IBD is considered a risk factor for several comorbid conditions, with patients showing a higher incidence of neurodegenerative diseases such as Alzheimer’s and Parkinson’s disease, as well as respiratory disorders like chronic obstructive pulmonary disease [6, 7].

Despite extensive research, the exact cause of IBD remains elusive, and it is believed to arise from a complex interplay of environmental, genetic, infectious and immune factors [8, 9]. Current treatment options for IBD include non-targeted therapies such as aminosalicylates, corticosteroids, and immunomodulators, as well as targeted therapies including anti-TNF, anti-IL-12/IL-23 agents, Janus kinase (JAK) inhibitors, and anti-integrin drugs [10, 11]. While many patients benefit from these targeted therapies, up to 30% do not respond initially, and as many as 50% experience delayed responses [12]. Additionally, many of these treatments are associated with significant side effects. Therefore, there is a critical need for new therapies to improve patient outcomes.

The establishment of animal models is essential for advancing our understanding of IBD pathogenesis and intestinal immune mechanisms, ultimately aiming to develop effective prevention and treatment strategies. Mice are particularly valuable due to their ability to closely replicate human intestinal pathology, making them a key tool for studying inflammation, immune responses, and treatment effects in IBD. However, mouse models have several limitations, including high costs, lengthy experimental timelines, and ethical considerations related to their use. To address these issues, alternative non-mammalian models, such as zebrafish (*Danio rerio*), fruit flies (*Drosophila melanogaster*), and nematodes (e.g., *Caenorhabditis elegans*), have also been developed [13, 14]. These models offer several advantages, including rapid development, genetic accessibility, and cost-effectiveness, while circumventing the ethical and practical challenges of mammalian research.

*D. melanogaster* is an excellent representative of non-mammalian models. Its small size, with a body length of only 3–4 mm, facilitates convenient laboratory maintenance.

Under standard conditions, it can develop from a newly laid egg to an adult in approximately 10 days [15]. Sexually mature females can lay 30–50 eggs per day, totaling over a thousand eggs throughout their lifespan [16]. The brief life cycle and extremely high reproductive capacity are significant advantages that can greatly accelerate experimental progress. Additionally, the development and application of specific gene knockout mutations, the Gal4/UAS system for gene expression control, CRISPR/Cas9 gene editing, and other tools have greatly enhanced the value of *D. melanogaster* as a model organism in molecular biology research [17].

Beyond these practical benefits, the fruit fly has a relatively simple yet highly conserved genome, with homologues of over 75% of human disease-related genes [18]. This genetic similarity, combined with the ease of generating transgenic lines and performing high-throughput genetic screens, makes the fruit fly an invaluable model for studying a range of human diseases, including tumors, diabetes, neurodegenerative diseases, kidney stones, and hyperuricemia [19–23].

**Figure 1.**
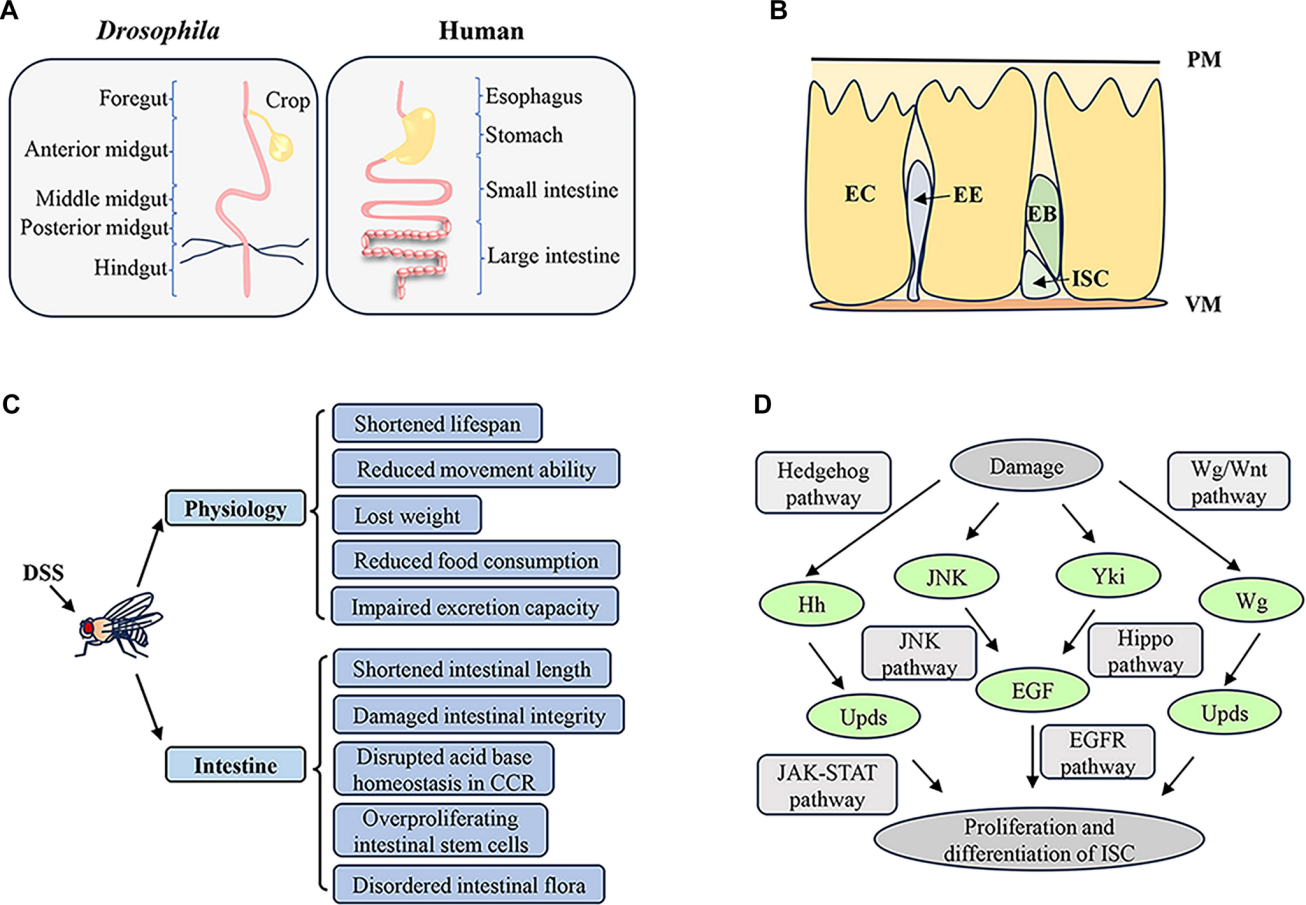
**Intestinal structure and IBD model of *D. melanogaster*.** (A) Comparison of intestinal anatomic structure between *D. melanogaster* and humans. The intestinal tract of *D. melanogaster* is divided into three main sections: The foregut, midgut, and hindgut. The midgut, which is analogous to the human small intestine, can be further subdivided into the anterior midgut, middle midgut, and posterior midgut. The hindgut serves a function comparable to that of the human colon. The crop in *D. melanogaster*, which acts as a temporary storage organ for food, can be considered functionally similar to the human stomach. (B) Intestinal cell composition of *D. melanogaster*. The cellular composition includes ISCs, ECs, EEs, and EBs. The asymmetric division of ISCs produces EBs, which eventually differentiate into ECs or EEs. VM, visceral muscle; PM, peritrophic matrix. (C) DSS-induced changes in physiological and intestinal state of *D. melanogaster*. From the perspective of physiological status, DSS intake resulted in shortened lifespan, reduced movement ability, weight loss, reduced food consumption, and impaired excretion capacity in *D. melanogaster*. In terms of intestinal health, DSS caused shortened intestinal length, damaged intestinal integrity, disrupted acid–base homeostasis in the intestinal copper cell region (CCR), overproliferation of ISCs, and disordered intestinal flora. (D) DSS-induced intestinal injury stimulates the proliferation and differentiation of ISCs through the modulation of several key signaling cascades. The primary pathways implicated in this response include the JAK/STAT, EGFR, JNK, Wnt/Wg, Hedgehog (Hh), and Hippo (Hpo) signaling pathways, which collaboratively govern the intricate process of ISC activation and intestinal repair. Abbreviations: IBD: Inflammatory bowel disease; ISC: Intestinal stem cell; JAK: Janus kinase; EC: Enterocyte; EE: Enteroendocrine cell; EB: Enteroblast; DSS: Dextran sulfate sodium; EGFR: Epidermal growth factor receptor.

The structural and functional parallels between the fruit fly gut and the human intestinal system (Figure 1A) further enhance its suitability for investigating intestinal diseases such as IBD [24]. *D. melanogaster* has served as a model organism for IBD research for over a decade, yielding significant insights [25]. This model has not only deepened our understanding of the biological processes involved in intestinal development and the maintenance of physiological functions but has also provided new perspectives and strategies for the treatment of IBD in humans.

This review summarizes recent advancements in using *D. melanogaster* as a model for IBD. It highlights various methods for modeling IBD in the fruit fly, with a particular focus on the widely used dextran sulfate sodium (DSS)-induced model. The review also discusses the proliferation and differentiation of intestinal stem cells (ISCs) during IBD, along with the signaling pathways involved in ISC differentiation, emphasizing the importance of gut microbiota in IBD pathogenesis and management. Finally, it categorizes anti-IBD compounds and drugs identified through the fruit fly model based on their mechanisms of action.

## Anatomic structure and cell composition of the fruit fly intestinal

The fruit fly gut is a tubular structure composed of a single layer of epithelial cells, divided into the foregut, midgut, and hindgut [26]. The foregut, which includes the esophagus, cardia, and crop [27], primarily functions in food ingestion. The crop is a sac-like structure that facilitates the mixing, detoxification, and storage of food [28]. The midgut, analogous to the human small intestine, plays key roles in digestion [29]. The hindgut is responsible for the reabsorption of water and ions, similar to the function of the human colon [30].

The midgut, developing from the endoderm, is further subdivided into the anterior midgut (AM), middle midgut (MM), and posterior midgut (PM). The AM expresses enzymes involved in the digestion of large molecules such as starch, lipids, and macroproteins [31]. Additionally, antimicrobial peptides (AMPs) are predominantly expressed in the AM compared to the posterior compartments, suggesting that the AM acts as the first defensive barrier [32]. The MM contains a pool of highly differentiated cells, including specialized cells called “copper cells,” which are functionally analogous to mammalian gastric parietal cells responsible for acid secretion. These cells secrete acid, creating an acidic environment known as the copper cell zone (CCR) [33]. This acidic environment is essential for processing certain molecules into monosaccharides, amino acids, and fatty acids, while also facilitating the reduction of metal ions [31]. The PM is the most metabolically active region of the gut in *D. melanogaster* [24]. It is specialized for the digestion and absorption of nutrients, allowing for the quick use or storage of small amounts of nutrients.

The cellular composition of the *D. melanogaster* midgut closely resembles that of mammals. The gut is mainly composed of four different cell types: ISCs, enterocytes (ECs), enteroendocrine cells (EEs), and enteroblasts (EBs) (Figure 1B). ECs are the fundamental structural component of the intestine, mainly responsible for secreting digestive enzymes and absorbing nutrients [29, 34, 35]. The apical surface of ECs is covered by microvilli, oriented towards the intestinal lumen, which increase the surface area and enhance nutrient absorption efficiency [36]. Additionally, the peritrophic membrane secreted by ECs serves a similar protective function to the lumen mucus secreted by mammalian goblet cells [37]. EEs are chemoreceptor cells that secrete hormone peptides, regulating intestinal movement and function in response to external stimuli [38, 39]. These hormone peptides facilitate interorgan communication, such as the gut-brain axis, relaying the organism’s nutritional status and influencing behavior and metabolism in a manner analogous to their mammalian counterparts [40, 41]. ISCs are the only cells in the gut that undergo mitosis, essential for replenishing damaged cells and maintaining intestinal self-renewal. ISCs can divide into new ISCs and EBs, with EBs further differentiating into ECs and EEs [42]. In mammals, intestinal homeostasis is similarly maintained by ISCs, which perform self-renewal by producing transient amplifying (TA) cells through mitosis [43].

## Establishment of a *D. melanogaster* model for IBD

Various methods have been developed to establish IBD animal models, with the oral administration of DSS being one of the most widely used. DSS, a polyanionic derivative of dextran with molecular weights ranging from 5 kDa to 1400 kDa, can be administered at varying concentrations, durations, and frequencies to induce acute, chronic, or recurrent inflammation [44]. This approach effectively mimics many pathological features of human ulcerative colitis in different animal models, including rat, mice, zebrafish, and *D. melanogaster* (Supplemental data), demonstrating characteristic manifestations such as hemorrhagic diarrhea, weight loss, colon shortening, mucosal ulcers, and neutrophil infiltration [1, 45, 46]. DSS models are particularly valued for their low cost, simplicity, and high reproducibility, making them the most used method to investigate the pathogenesis of IBD, explore therapeutic mechanisms, and evaluate potential treatments.

Although DSS is widely used in IBD research, its pathogenic mechanism remains not fully understood. It is generally believed that DSS exerts direct toxic effects on intestinal epithelial cells, leading to epithelial damage and compromised barrier integrity. This disruption allows intestinal bacteria and related antigens to penetrate the mucosa, triggering an inflammatory immune response [47–49]. Laroui et al. [50] discovered that DSS forms nanometer-sized vesicles by complexing with medium-chain length fatty acids (MCFAs).

These vesicles fuse with colonocyte membranes, disrupting the intestinal barrier and initiating the inflammatory signaling cascade. Additionally, it has been suggested that DSS chelates divalent cations, such as Ca^2+^ and Mg^2+^, leading to the loss of the tight junction protein ZO-1. This results in disruption of intercellular tight junctions and increased epithelial permeability, thereby contributing to the onset of intestinal inflammation [51, 52]. Notably, the resulting inflammatory environment further worsens barrier dysfunction by triggering the release of pro-inflammatory cytokines and promoting epithelial cell death. This creates a self-sustaining cycle of barrier damage and inflammation. Although this mutual relationship is well recognized, the detailed molecular mechanisms and timing of events involved in this feedback loop are still not fully understood, pointing to a key area for future research [53].

Oral administration of DSS induces enteritis in various animals, including rats, zebrafish, and fruit flies, resulting in similar symptoms. In *D. melanogaster*, DSS disrupts midgut functions, leading to inflammation, microbiota disturbance, loss of intestinal integrity, and alterations in the midgut’s acid-base balance. This disruption results in reduced food intake, impaired excretion, weight loss, and shortened lifespan in the flies (Figure 1C) [1, 54]. Additionally, DSS-induced inflammation accelerates the differentiation and proliferation of ISCs [55]. The symptoms mirror those seen in mammalian models, confirming that DSS can induce an IBD-like phenotype in fruit flies, making it a reliable model for IBD research.

In addition to oral administration of DSS, several other methods can induce IBD in *D. melanogaster.* For instance, feeding sodium dodecyl sulfate (SDS) or bleomycin damages intestinal cells and mimics IBD by triggering inflammation [42, 56]. Inducing oxidative stress with paraquat also leads to intestinal inflammation [57]. Moreover, the oral administration of pathogenic bacteria such as *Pseudomonas aeruginosa* and *Erwinia carotovora carotovora 15* (Ecc15) can cause apoptosis of mature cells and increase the number of ISCs, establishing models of intestinal infection and injury [55, 58]. Sleep deprivation, which generates peroxides that trigger gut inflammation, is also emerging as a novel modeling method for IBD [59].

## Inflammation in the DSS model

DSS administration induces various inflammatory signaling pathways in the *D. melanogaster* gut, which share certain similarities with the mammalian intestinal inflammatory response. DSS can induce significant damage to both the intestinal mucosa and tight junction complexes, leading to compromised gut barrier integrity. This pathological alteration triggers the upregulation of pro-inflammatory factors such as Unpaireds (Upds), homologous to mammalian interleukins (ILs). This upregulation activates the JNK pathway and the JAK/STAT pathway, resulting in excessive proliferation and differentiation of ISCs [60, 61]. Additionally, DSS intake triggers the activation of DUOX, a member of the NADPH oxidase family, leading to massive reactive oxygen species (ROS) production [62]. This ROS production promotes oxidative stress and innate immune system responses, activating the NFκB signaling pathway [63]. Consequently, this activation causes intestinal flora disturbance and changes in microbial composition, with DUOX playing a key role in intestinal defense in *D. melanogaster*.

## Proliferation and differentiation of ISCs under the DSS model

In *D. melanogaster*, the midgut contains numerous ISCs crucial for maintaining epithelial function. ISC proliferation can increase in response to injury, thereby promoting regeneration and gut repair [55, 64]. However, excessive proliferation without proper differentiation causes dysplasia, exacerbates inflammation and can even leads to cancer [65, 66]. ISCs can divide through two distinct mechanisms: asymmetric and symmetric division. Asymmetric division results in the formation of one ISC and one EB, which further differentiates into either ECs or EEs. This process ensures the continual renewal of the gut epithelium by producing cells necessary for nutrient absorption and hormonal regulation. In contrast, symmetric division can produce either two identical ISCs or two identical EBs, allowing for either stem cell self-renewal or expansion of the progenitor cell pool [67–70].

Key markers are used to identify these cell types. Escargot, a member of the SNAIL family of transcription factors, is expressed exclusively in ISCs and EBs, serving as a critical marker for these undifferentiated cells [71]. Prospero, a homeodomain protein, is specifically found in EEs and is vital for their differentiation and function [55]. Additionally, Pdm1, a Class II POU domain transcription factor, is expressed only in mature ECs [72].

In the *D. melanogaster* IBD model induced by DSS, intestinal cells exhibit abnormal changes. DSS leads to excessive proliferation of ISCs, accumulation of EBs, and differentiation of EEs without inducing EC differentiation. This process is regulated by several signaling pathways, including JAK/STAT, epidermal growth factor receptor (EGFR), JNK, Wnt/Wg, Hedgehog (Hh), and Hippo (Hpo), which work together to maintain intestinal homeostasis (Figure 1D).

### JAK-STAT pathway

The JAK/STAT pathway is a conserved signal transduction pathway involved in cell growth, differentiation, apoptosis, and immune regulation. In *D. melanogaster*, there are three IL-6-like cytokines known as Unpaireds (Upd, Upd2, Upd3) [73]. These cytokines bind to the receptor Domeless (Dome), thereby promoting activation of the JAK called Hopscotch (Hop) and STAT transcription factors, which subsequently regulate gene expression [74]. When the intestinal tract of *D. melanogaster* is damaged by DSS ingestion, ECs produce high levels of Unpaireds [75, 76]. This triggers JAK/STAT signaling in ISCs and EBs, promoting their division and differentiation, and driving regeneration and renewal of the intestinal epithelium. Knockdown of STAT or Dome results in loss of JAK/STAT signaling and inhibits injury-induced tissue regeneration [77].

### EGFR pathway

In *D. melanogaster*, various cellular processes—including cell survival, proliferation, differentiation, and migration—rely on EGFR signaling [78]. The pathway is activated by epidermal growth factor (EGF) ligands, such as Vein, Spitz, and Keren, which are expressed in visceral muscle (VM), progenitor cells (EBs and ISCs), and ECs, respectively [79]. Vein is produced as a secreted protein and does not require further processing, whereas Spitz and Keren are membrane-bound precursor proteins that must bind to the chaperone protein STAR to form a complex [80]. This complex is then cleaved by the protease Rhomboid, activating the EGFR pathway and promoting ISC proliferation. In the presence of DSS, damage to the basement membrane and ECs in the *D. melanogaster* midgut further induces the expression of EGF and fly cytokines in the intestine. This activates both the EGFR and JAK/STAT pathways, resulting in excessive proliferation of ISCs [81–84]. When a dominant-negative form of EGFR (EGFRˆDN) is overexpressed, the increase in ISC numbers is no longer significant, indicating the critical role of EGFR signaling in this process [54].

### JNK pathway

The JNK signaling pathway, an important branch of the mitogen-activated protein kinase (MAPK) pathway [85, 86], plays a crucial role in various physiological and pathological processes, such as cell stress, regeneration, apoptosis, and immunity [86]. In mammals, three JNK genes are present, while *D. melanogaster* has a single JNK gene known as basket (bsk), simplifying genetic analysis [87]. Following intestinal damage in *D. melanogaster*, JNK is activated in both ISCs and ECs. Its activation in ISCs induces their proliferation through phosphorylation of the AP-1 transcription factor Fos. In ECs, JNK activation stimulates the production of Upds, leading to ISC proliferation [88]. Additionally, JNK activation in ECs triggers activation of the JAK/STAT pathway and expression of EGF ligands, further promoting ISC proliferation through EGFR pathway activation [89].

### Wnt/Wg pathway

As an evolutionarily conserved signaling pathway, the Wnt/Wg pathway profoundly impacts embryonic development, tissue regeneration, stem cell maintenance, and other processes. In *D. melanogaster*, the Wingless protein (Wg) secreted by EBs binds to the Frizzled (Fz) receptor and Arrow (Arr) co-receptor, initiating downstream signaling cascades. This interaction leads to accumulation of the key downstream effector Armadillo (Arm), which translocates to the nucleus and binds to the nuclear transcription factor Pangolin (Pan). This binding drives expression of target genes involved in ISC division or maintenance [90, 91]. In the DSS model, intestinal epithelial injury upregulates Wg expression, activating the Wnt/Wg pathway and promoting ISC proliferation and tissue regeneration [92]. When Wg or fz genes are knocked out, ISC self-renewal is significantly impaired, underscoring the critical role of Wnt/Wg signaling in maintaining ISC homeostasis and regeneration [90].

### Hedgehog pathway

The Hedgehog (Hh) pathway is essential for ISC proliferation. Aberrant activation of Hh signaling promotes excessive ISC proliferation and disrupts tissue homeostasis [93]. The Hh signaling molecule is a localized protein ligand secreted by signaling cells, which binds to its transmembrane receptor Patched (Ptc) [91]. This binding relieves the inhibitory effect of Ptc on another transmembrane protein, Smoothened (Smo) [94]. Consequently, Smo activation promotes the transcription factor Cubitus interruptus (Ci) to translocate to the nucleus, initiating expression of related genes [95]. Although Hh signaling is not required for basal ISC maintenance, it is critically recruited during tissue repair to drive proliferative responses [96]. In *D. melanogaster*, damage induces an increase in Hh signaling in EBs, promoting ISC proliferation [97]. Blocking Hh signaling by knocking down smo in EBs—but not in ISCs—inhibits DSS-induced ISC proliferation. Hh signaling in EBs promotes ISC proliferation by regulating upd2 production, which subsequently activates the JAK/STAT pathway in ISCs, driving their proliferation. Additionally, the JNK pathway is necessary for damage-induced Hh pathway activation; inhibition of the JNK pathway blocks DSS-induced Hh upregulation in EBs and inhibits excessive ISC proliferation [97].

### Hippo pathway

The Hippo (Hpo) pathway, first discovered in *D. melanogaster*, plays a crucial role in regulating stem cell self-renewal and tissue regeneration [98]. The core components of this pathway include the Hpo kinase and its binding partner Sav, the Wts kinase and its binding partner Mats, and the transcription coactivator Yorkie (Yki, homologous to mammalian Yap) [99]. In the Hippo pathway, the Hpo–Sav complex phosphorylates and activates the Wts–Mats complex [100–105], which then phosphorylates the downstream Yki [106], restricting its activity and preventing its entry into the nucleus, thereby regulating tissue growth. During DSS-induced intestinal injury, Yki becomes activated and translocates to the nucleus, where it forms a complex with the transcription factor Scalloped (Sd) [106–109]. This complex activates genes involved in cell proliferation, growth, and apoptosis. Studies have shown that Yki is required in precursor cells for DSS-induced ISC proliferation. Specifically, knocking down yki using RNA interference (RNAi) in ISCs and EBs—but not in ECs—suppresses DSS-induced ISC proliferation [110]. Moreover, Yki activation leads to increased expression of Upds and multiple EGF ligands [99]. These molecules activate the JAK/STAT and EGFR signaling pathways in ISCs, promoting stem cell proliferation in a cell non-autonomous manner. As a downstream effector of the Hpo pathway, dMyc also plays a role in regulating ISC proliferation. Knockdown of dMyc suppresses DSS-induced ISC proliferation [111].

## Intestinal flora and immunity

The gut provides an optimal environment for microbes to thrive. Under normal conditions, the intestinal flora maintains a state of microecological equilibrium, fostering a mutually beneficial and symbiotic relationship with the host. The host provides a suitable living environment for the microbiota, which in turn contributes to various physiological processes, including digestion and metabolism, pathogen defense, regulation of the intestinal barrier, and modulation of the immune system [112]. Typically, the host’s immune response effectively eliminates pathogens that enter the gut through food ingestion. The immune response in the gut primarily relies on the production of local ROS and the release of AMPs [113]. However, when the immune system is compromised, this balance is disrupted, leading to an inflammatory response and gut microbial dysregulation [114].

ROS serve as the primary line of defense in intestinal immunity and can be generated by the transmembrane protein dual oxidase (DUOX). As a member of the NADPH oxidase family, the expression and activity of DUOX are both regulated in response to changes in the intestinal microbiota. During inflammation, DUOX is activated to generate substantial amounts of ROS, aiding the host in defending against external stressors and pathogenic infections [115]. However, excessive ROS production can also lead to detrimental effects, including oxidative stress, disruption of intestinal cell structure and function, damage to the intestinal mucosal barrier, and activation of pro-inflammatory signaling pathways, thereby exacerbating intestinal injury [116, 117]. The secretion of AMPs relies on the NFκB pathway, which comprises the Toll and Imd pathways [118]. The Toll pathway primarily protects against fungi and Gram-positive bacteria, while the Imd pathway is chiefly responsible for defending against Gram-negative bacteria [119]. The Toll signaling pathway was initially discovered in *D. melanogaster* [120]. The Toll receptor is a transmembrane protein that, during an immune response, recognizes and binds to a specific ligand called Spätzle, triggering its activation. This activation enables NF-κB-like transcription factors Dif and Dorsal to enter the nucleus and induce AMP production [121, 122]. The Imd pathway, on the other hand, is activated upon recognition of the peptidoglycan (PGN) in the cell walls of Gram-negative bacteria by the transmembrane receptor PGRP-LC and the intracellular receptor PGRP-LE [123]. This recognition activates the Imd pathway, leading to the transfer of the N-terminal of the NFκB transcription factor Relish into the nucleus, where it promotes the transcription of AMP genes (Attacin A, Cecropin C, Defensin, and Diptericin) that are essential for bacterial clearance [124, 125].

Compared to vertebrates, the microbial diversity in the intestinal tract of *D. melanogaster* is relatively low, typically encompassing only 5–30 species, with Lactobacilli and Acetobacter being the most prevalent [126]. At the phylum level, the primary microorganisms found in the gut of *D. melanogaster* include Proteobacteria, Firmicutes, Actinobacteria, Bacteroidetes, and Acidobacteria [127]. In the DSS-induced IBD model, the flora of *D. melanogaster* becomes dysregulated, resulting in a decrease in microbial diversity, an increase in the relative abundance of Firmicutes, and a decrease in the relative abundance of Proteobacteria and Actinobacteria [1, 127]. This dysregulation triggers an immune response and inflammation, resulting in activation of the NFκB pathway. Notably, the NFκB transcription factor Relish was not activated when DSS was administered to sterile fruit flies, suggesting that the activation of immune pathways induced by DSS is influenced by the disordered intestinal flora [128].

Additionally, Zhang et al. found that bilberry anthocyanin extract could induce changes in gut microbiota and reduce inflammation in DSS-induced *D. melanogaster* models. Furthermore, they observed that this relief from inflammation was consistent in both conventional and germ-free *D. melanogaster*, suggesting that gut microbiota was not involved in the alleviation of intestinal inflammation [127]. However, research has demonstrated that Myroides pelagicus, obtained from the gut of *D. melanogaster*, exhibits therapeutic efficacy in DSS-induced mouse models [129]. Thus, the role of beneficial gut microbiota in mitigating inflammation remains a subject of debate.

There is no doubt that intestinal microbiota dysbiosis is a significant characteristic of IBD, presenting a valuable avenue for IBD treatment and drug development. However, current evidence remains inconclusive regarding both the mechanisms by which beneficial bacteria regulate gut immune homeostasis and the potential causative links between microbial dysbiosis and IBD, highlighting the need for systematic investigation in future research. A systematic and in-depth analysis of these scientific issues is crucial for developing effective therapeutic strategies that target both gut microbiota dysbiosis and IBD.

## Anti-IBD ingredients and drugs

The fruit fly IBD model has also been employed to identify active compounds and drugs for the treatment of IBD, as well as to investigate their therapeutic mechanisms. Most of the results are consistent with the conclusions of studies on mammalian models and clinical studies and can mutually confirm each other (Table 1). In the in-depth mechanism research based on the fruit fly model, the therapeutic mechanisms can be categorized into several key areas: the regulation of inflammation-associated signaling pathways, modulation of oxidative stress, immune modulation, regulation of intestinal flora, and autophagy regulation.

**Table 1:** Anti-IBD activity of natural products

**Compound**	**Model**	**Mechanism**	**Reference**	**Consistency with mammalian models and clinical trial outcomes**
San Huang Pill	DSS model	Regulating JAK/STAT, Toll, Nrf2/Keap1 pathways, apoptosis, and intestinal microflora	[76]	
Premna microphylla turcz polysaccharid	SDS model	Regulating intestinal microflora and immune response	[140]	[146]
Stilbenoid compounds-pinosylvin	DSS model	Regulating immune response	[128]	[147]
Stilbenoid compounds-pinosylvin monomethyl ether	DSS model	Regulating immune response	[128]	
Astragalus membranaceus extract	SDS model	Regulating JNK and JAK/STAT pathways	[56]	[148]
Acanthopanax senticosus polysaccharide	SDS and DSS model	Regulating EGFR, JNK and Notch pathways	[130]	[149]
Allomyrina dichotoma larval extract	DSS model	Enhancing E-cad expression and preserves its membrane localization with arm	[150]	[151]
Silibinin	DSS model	Regulating JNK pathway	[54]	[152]
Bilberry anthocyanin extracts	DSS model	Regulating Nrf 2 pathway	[127]	[153]
Orostachys malacophylla (Pall.) Fisch extract	DSS and Ecc15 model	Regulating oxidative stress and immune response	[154]	
Extracts of hylotelephium erythrostictum (Miq.) H. Ohba	DSS and Ecc15 model	Regulating JNK, EGFR, JAK/STAT pathways and oxidative stress	[134]	
Carrageenan oligosaccharide	SDS model	Regulating intestinal microflora and IMD/relish pathway	[137]	[155]
Flos puerariae extract	SDS model	Regulating Nrf2/Keap1, JAK/STAT and Wnt pathways	[132]	[156]
Total ginsenosides	SDS model	Regulating MAPK pathway	[133]	[157]
Chitosan oligosaccharide	H_2_O_2_ model	Regulating autophagy, intestinal microflora and the antioxidant signaling pathways	[138]	[158]
Ursolic acid	SDS model	Regulating JNK pathway	[131]	[159]
Safranal	DSS and Ecc15 model	Regulating JNK, EGFR and JAK/STAT pathways	[58]	[160]
Codonopsis pilosula (Franch.) Nannf	SDS model	Regulating IMD pathway	[139]	[161]
Agar oligosaccharide	SDS model	Regulating autophagy, intestinal microflora and immune response	[125]	[162]

Abbreviations: IBD: Inflammatory bowel disease; JAK: Janus kinase; DSS: Dextran sulfate sodium; SDS: Sodium dodecyl sulfate; Ecc15: Erwinia carotovora carotovora 15; EGFR: Epidermal growth factor receptor; MAPK: Mitogen-activated protein kinase.

### Regulation of inflammation-associated signaling pathways

IBD patients exhibit severe intestinal inflammation, believed to originate from abnormalities in relevant signaling pathways. Treating IBD with targeted drugs that regulate these inflammation-associated signaling pathways is considered a promising strategy.

In the DSS-induced IBD model, a variety of drugs and components have been found to play a therapeutic role by regulating inflammation-related signaling pathways. San Huang Pill, a prescription from Dunhuang Ancient Medical Prescriptions, which consists of Coptis chinensis Franch, Scutellaria baicalensis Georgi, and Rheum palmatum L at a ratio of 1:1:1, has been shown to alleviate intestinal damage by inhibiting the JAK/STAT pathway [76]. Polysaccharides from Acanthopanax senticosus significantly improve the survival rate of *D. melanogaster* by regulating EGFR, JNK, and Notch signaling pathways, ultimately reducing the excessive proliferation and differentiation of ISCs to achieve the anti-IBD effect [130]. Silybin, derived from the seeds of milk thistle (Silybum marianum (L.) Gaertn.), inhibits excessive ISC proliferation and alleviates intestinal inflammation by regulating the JNK signaling pathway [54]. Similar examples were also found in SDS-induced models. Both Astragalus membranous extract and ursolic acid reduce intestinal inflammation by inhibiting the JNK and JAK-STAT signaling pathways [56, 131]. The extract of flos puerariae may prevent intestinal damage by inhibiting JAK-STAT and Wnt signaling pathways [132]. Total ginsenosides, the active substances of ginseng (Panax ginseng), can improve the survival rate and climbing ability of *D. melanogaster* and repair intestinal damage by regulating the MAPK signaling pathway, suggesting their potential application in the treatment of IBD [133]. Both safranal and the extracts of Hylotelephium erythrostictum (Miq.) H. Ohba mitigate ISC hyperproliferation and differentiation by inhibiting the JNK, EGFR, and JAK/STAT pathways [58, 134]. These exert anti-IBD effects in models induced by Ecc15 and DSS.

### Modulation of oxidative stress

The overproduction of ROS and the consequent oxidative stress play a key role in the pathophysiology of IBD. The protective effects observed from exogenous antioxidants and the transgenic overexpression of antioxidant genes in IBD models underscore the significant involvement of oxidative stress in the disease’s progression. For example, intravenous administration of the antioxidant lecithin superoxide dismutase has been shown to be safe and effective in improving the clinical condition of patients with active ulcerative colitis [135]. In a mouse model of DSS-induced colitis, treatment with N-acetylcysteine (NAC) improves intestinal mucosal glutathione (GSH) levels and prevents histological damage to the colonic mucosa [136]. Furthermore, the overexpression of copper (Cu) and zinc superoxide dismutase (ZnSOD) also alleviates DSS-induced colitis in mice [135]. Similarly, in the *D. melanogaster* model, the regulation of oxidative stress is also an important therapeutic mechanism. The addition of carrageenan oligosaccharides to the diet significantly reduces ROS levels in the midgut of fruit flies treated with SDS-H_2_O_2_ [137]. This reduction is attributed to increased activities of superoxide dismutase (SOD) and catalase (CAT), which are responsible for ROS clearance, as well as decreased malondialdehyde (MDA) levels. The Nrf2 pathway is a critical regulator of oxidative stress, making it a promising target for antioxidant-based interventions in IBD. For example, San Huang Pill significantly lowers ROS levels in the gut while upregulating the expression of Nrf2 pathway genes, including CncC, Keap1, sod1, sod2, and cat, thereby alleviating DSS-induced intestinal oxidative damage [76]. Extracts of Chinese medicinal materials such as bilberry anthocyanin extract and flos puerariae extract can also protect the intestine by acting on the Nrf2 pathway [127, 132]. Marine functional chitosan oligosaccharide can promote the activation of the Nrf2 pathway, increase CAT activity, and decrease MDA content, thereby improving the antioxidant capacity of *D. melanogaster* and maintaining its homeostasis [138].

### Immune modulation

In patients with IBD, the intestinal immune system exhibits an overactive response, resulting in inflammation and tissue damage. Consequently, regulating the immune response is crucial for effective IBD treatment.

San Huang Pill significantly reduces the expression levels of genes related to the Toll-signaling pathway, thereby regulating the immune response [76]. The mechanisms by which carrageenan oligosaccharides and chitosan oligosaccharide relieve intestinal inflammation depend on the IMD signaling pathway [137, 138]. Aqueous extracts from Codonopsis pilosula, agar oligosaccharide, and Premna microphylla Turcz. polysaccharide can induce the expression of AMP genes to enhance the immune response and prolong the life of SDS-stimulated *D. melanogaster* [139, 140]. Stilbene compounds pinosylvin and pinosylvin monomethyl ether inhibit transient receptor potential ankyrin 1 (TrpA1) channels, indirectly regulate DSS-induced Relish activation, and exert anti-inflammatory effects [128].

### Regulation of intestinal flora

Intestinal flora plays an important role in maintaining intestinal function, homeostasis, and immune regulation. A fundamental characteristic of IBD patients is the disruption of intestinal flora. Therefore, regulating intestinal flora and restoring it to homeostasis is a crucial component of IBD treatment strategies. The sequencing results of the 16S rRNA gene showed that San Huang Pill protects the gut by reducing the relative abundance of harmful bacteria in fruit flies induced by DSS [76]. Premna microphylla Turcz. polysaccharide, carrageenan oligosaccharides, agar oligosaccharide, and chitosan oligosaccharide can reduce inflammation by increasing intestinal microbial diversity and regulating intestinal microbial composition [125, 137, 138, 140]. This regulation plays a significant role in improving physiological symptoms such as longevity and intestinal integrity in *D. melanogaster*.

### Regulation of autophagy

Autophagy is an internal cellular degradation and recycling process that removes damaged or unnecessary intracellular components to maintain a stable internal environment [141]. In the IBD model, damage to intestinal cells can trigger autophagy. However, under inflammatory stimulation, autophagy may become over-activated, resulting in excessive degradation of intracellular components and further exacerbating cell damage and death. Studies have shown that both agar oligosaccharide and chitosan oligosaccharide can reduce the expression of autophagy-related genes (AMPKα, Atg1, Atg5, and Atg8a) to mitigate excessive autophagy in the intestine, thereby alleviating intestinal injury [125, 138]. In contrast, Song et al. found that Premna microphylla Turcz. polysaccharide can increase the expression of autophagy-related genes and plays a therapeutic role by stimulating intestinal autophagy [140].

## Conclusion

In summary, the utilization of *D. melanogaster* as a model for studying IBD has provided valuable insights into the complex interplay between the gut microbiota, immune responses, ISC dynamics, and the pathophysiological mechanisms underlying IBD. The *D. melanogaster* model presents distinct advantages, including minimal ethical concerns, higher experimental efficiency, lower costs, and a versatile genetic toolkit, enabling more profound scientific exploration. The findings emphasize the role of ISCs in intestinal regeneration and their potential dysregulation during inflammation, which can contribute to disease progression. Furthermore, various natural compounds, including traditional herbal medicines, have shown promise in modulating inflammation, oxidative stress, and autophagy, thus alleviating intestinal injury and supporting ISC homeostasis.

Moving forward, future research should focus on elucidating the causal relationships between dysbiosis and IBD, particularly how changes in microbial composition influence ISC behavior and intestinal repair mechanisms. Further investigations should also explore the protective role of beneficial microbiota in maintaining intestinal homeostasis, as well as the disruptive effects of pathogenic bacteria on gut barrier integrity and immune regulation. Understanding the interaction between inflammation, ISCs, and gut microbiota could lead to innovative therapeutic strategies that target microbial imbalances alongside inflammatory pathways and ISC dysregulation. Notably, *D. melanogaster* aging exhibits intestinal barrier dysfunction and microbial dysbiosis, which phenotypically resemble IBD symptoms [142–145]. However, whether these age-related changes can be directly equated with IBD, and whether IBD treatment mechanisms are equivalent to longevity-promoting mechanisms, requires further investigation. Additionally, leveraging advanced genomic and transcriptomic techniques may enhance our ability to identify and validate new therapeutic targets within the context of the gut microbiome, innate immunity, and ISC biology. Ultimately, these efforts hold promise for the development of more effective and personalized treatment options for patients suffering from IBD.

## Supplemental data

**Graphical abstract.**
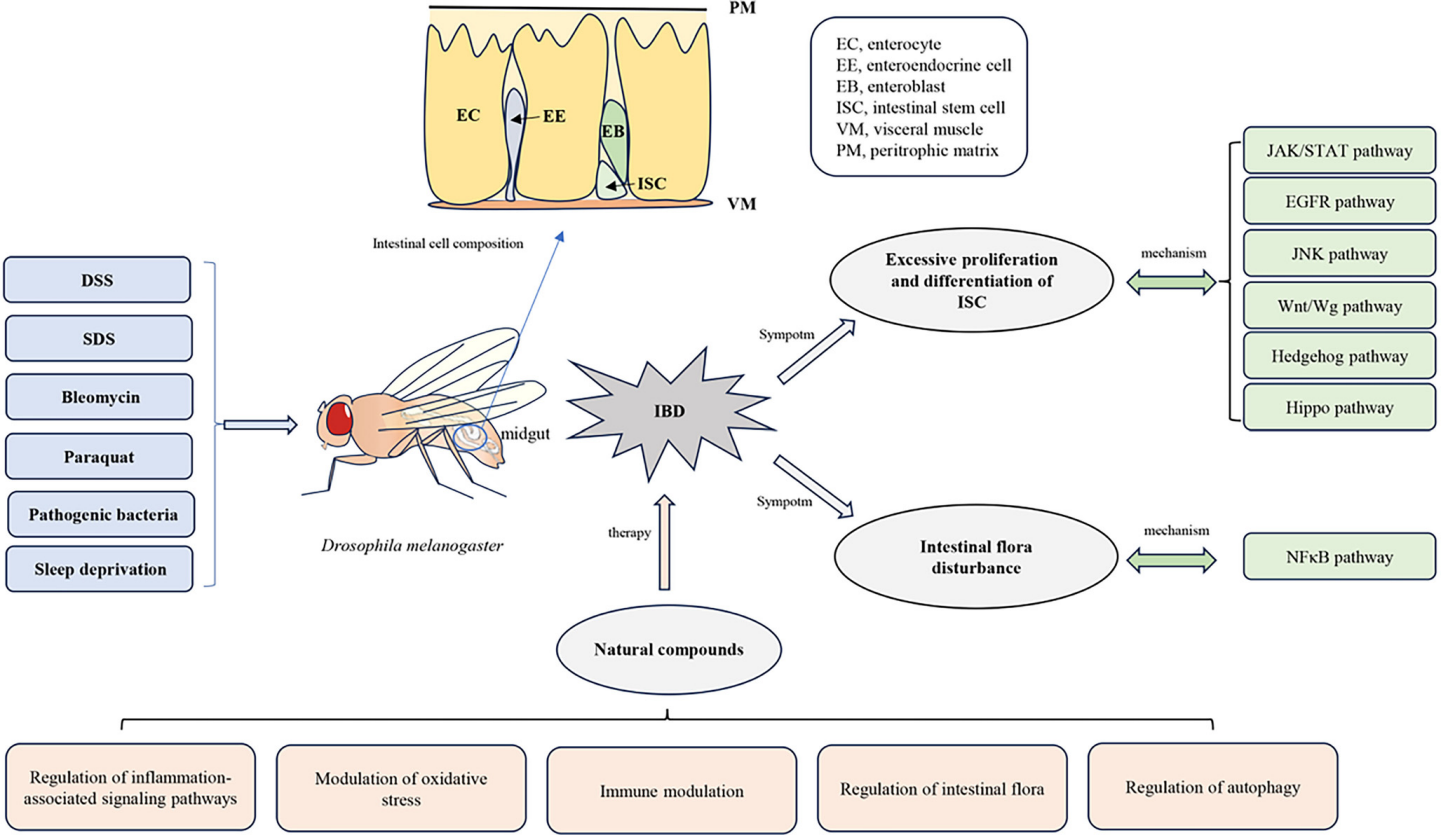
**Establishment methods of the D. melanogaster IBD model and a summary of physiological phenotypes related to the IBD model.** Abbreviations: IBD: Inflammatory bowel disease; ISC: Intestinal stem cell; JAK: Janus kinase; DSS: Dextran sulfate sodium; SDS: Sodium dodecyl sulfate; EGFR: Epidermal growth factor receptor.
